# Equine Influenza A(H3N8) Virus Isolated from Bactrian Camel, Mongolia

**DOI:** 10.3201/eid2012.140435

**Published:** 2014-12

**Authors:** Myagmarsukh Yondon, Batsukh Zayat, Martha I. Nelson, Gary L. Heil, Benjamin D. Anderson, Xudong Lin, Rebecca A. Halpin, Pamela P. McKenzie, Sarah K. White, David E. Wentworth, Gregory C. Gray

**Affiliations:** Institute of Veterinary Medicine, Ulaanbaatar, Mongolia (M. Yondon, B. Zayat);; National Institutes of Health, Bethesda, Maryland, USA (M.I. Nelson);; University of Florida, Gainesville, Florida, USA (G.L. Heil, B.D. Anderson, S.K. White, G.C. Gray);; J. Craig Venter Institute, Rockville, Maryland, USA (X. Lin, R.A. Halpin, D.E. Wentworth);; St. Jude Children’s Research Hospital, Memphis, Tennessee, USA (P.P. McKenzie)

**Keywords:** influenza A virus, Mongolia, interspecies transmission, *Camelus bactrianus*, Bactrian camel, viruses, influenza

## Abstract

Because little is known about the ecology of influenza viruses in camels, 460 nasal swab specimens were collected from healthy (no overt illness) Bactrian camels in Mongolia during 2012. One specimen was positive for influenza A virus (A/camel/Mongolia/335/2012[H3N8]), which is phylogenetically related to equine influenza A(H3N8) viruses and probably represents natural horse-to-camel transmission.

Since the first isolation in 1963 of an avian-origin influenza A(H3N8) virus from horses (*1*), subtype H3N8 influenza viruses have continued to circulate panzootically among horses, causing severe outbreaks of equine influenza respiratory disease. In the United States, these viruses jumped from horses to dogs and continue to circulate among dogs (*2*,*3*). In Mongolia, the site of some of the world’s largest epizootics of equine influenza A(H3N8) virus (EIV) infection, transmission of this virus is sustained among 2.1 million free-ranging horses, causing significant economic losses among rural herders. Major epizootics of EIV infection occurred in Mongolia during 2007–2008 (459,000 cases, 24,600 deaths) and again in 2011 (74,608 cases, 40 deaths) (*4*).

Over previous decades in Mongolia, outbreaks of respiratory disease, thought to be influenza, among camels have been reported. In the 1980s, the virus was characterized, and researchers speculated that it was related to a reassortant influenza A(H1N1) virus vaccine strain, A/PR-8/34 + A/USSR/77, generated in a Soviet laboratory and administered to humans in Mongolia and possibly transmitted from vaccinated humans to camels in a reactivated form (*5*,*6*). However, only 1 genetic sequence from this outbreak among camels is available in GenBank: A/camel/Mongolia/1982/H1N1. Despite reports of serologic activity against influenza A virus among camels in several African countries (*7*,*8*), the lack of isolated virus from these populations highlights how little is known about the ecology of influenza viruses in camels. Questions about the potential role of camels in human cases of Middle East respiratory syndrome (*9*) further highlight our lack of knowledge of infectious diseases in camels and the merits of increased surveillance at this unique human–animal interface.

Since January 2011, surveillance of equine influenza viruses has been enhanced in 3 Mongolian aimags (provinces). Surveillance among camels was also initiated in response to anecdotal reports of signs of respiratory illness in Bactrian camels (*Camelus bactrianus*). We describe the isolation, full-genome sequencing, and phylogenetic characterization of an influenza A(H3N8) virus of equine lineage isolated from a Bactrian camel, thereby identifying a novel route of influenza virus interspecies transmission and raising further questions about influenza A virus ecology in under-studied regions such as Mongolia.

## The Study

**Figure 1:**
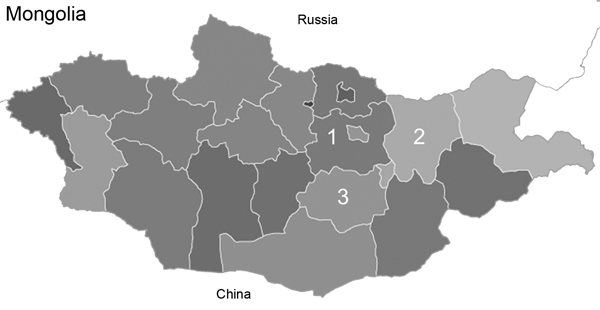
The 3 aimags from which nasal swab specimens were collected from healthy Bactrian camels, for influenza A virus testing, Mongolia, 2012. 1, Töv; 2, Khentii; 3, Dundgovi.

During January 2012–January 2013, a total of 460 nasal swab specimens were collected through active surveillance of horses and camels in 3 Mongolian aimags (Figure 1) known for high densities of free-ranging horses and camels (Table). Specimens were collected monthly, as weather permitted, from 50 free-ranging horses and 20 free-ranging Bactrian camels that were safely and carefully restrained with halters, ropes, and by hand, according to a protocol approved by the Department of Veterinary and Animal Breeding, Government of Mongolia. During sampling, camels were in a crouched or take-down position. Horse and camel specimens were carefully stored and shipped in separate containers; to prevent cross-contamination with EIV, specimens were separated during laboratory analyses.

**Table:** Number of specimens collected from camels, by aimag, each month, and result of testing for influenza A virus, Mongolia, January 2012–January 2013*

Date	Aimag (province)							
	Töv			Khentii			Dundgovi	
	Positive	Total		Positive	Total		Positive	Total
2012								
January	0	20		–	–		–	–
February	–	–		–	–		0	20
March	0	20		–	–		–	–
April	0	20		0	20		–	–
May	0	20		–	–		0	20
June	0	20		–	–		0	20
July	–	–		0	20		0	20
August	0	20		–	–		0	20
September	0	20		–	–		0	20
October	0	20		0	20		–	–
November	–	–		0	20		4	20
December	0	20		–	–		0	20
2013								
January	2	20		0	20		–	–
Total	2	200		0	100		4	160

*Testing was performed by quantitative reverse transcription PCR. A total of 460 specimens were collected across all aimags. – denotes when sampling did not occur because of poor weather or road conditions.

All specimens were first screened at the Institute of Veterinary Medicine laboratory (Ulaanbaatar, Mongolia) by using the World Health Organization influenza A quantitative reverse transcription PCR (qRT-PCR) protocol (*10*). Six specimens collected from camels without respiratory signs were positive for influenza A virus and were double-blind passaged in embryonated chicken eggs. Subsequent testing revealed hemagglutination activity in all 6 specimens. Allantoic fluid of the 6 cultured specimens was then shipped to the University of Florida for confirmation testing and sequencing. Only 1 specimen was confirmed positive by influenza A virus–specific qRT-PCR (cycle threshold [C_t_]<35), suggesting possible virus degradation during shipment, despite specimens being shipped on dry ice and carefully handled upon receipt (*10*). The original swab specimen from a camel was later shared with the Mongolia National Influenza Center for confirmation in an anonymized panel of 10 camel swab specimens. Using World Health Organization qRT-PCR procedures, staff identified the specimen as having the strongest evidence (by C_t_) of influenza A virus. Staff further studied the specimen with conventional RT-PCR primers and probes for the hemagglutinin and neuraminidase genomes. These reactions yielded amplicons of the expected size, which were sequenced and found to be 100% identical to the corresponding portions of the J. Craig Venter Institute (Rockville, MD, USA) sequences described below.

Sanger sequence data for the hemagglutinin and neuraminidase genes of this isolate demonstrated extremely high levels of identity with recent EIV from Asia isolated under this project in 2011 (*4*). Full-genome sequencing of the 8 genome segments amplified by multisegment RT-PCR (*11*) was performed at the J. Craig Venter Institute by using Ion Torrent PGM technology (Life Technologies, Grand Island, NY, USA) with a 314v2 chip, and the sequences were validated by using the MiSeq platform (Illumina, Inc., San Diego, CA, USA). Full-genome sequence data for this virus, named A/camel/Mongolia/335/2012(H3N8), were deposited in GenBank (accession nos. CY164120.1, CY164121.1, CY164122.1, CY164123.1, CY164124.1, CY164125.1, CY164126.1 and CY164127.1). The isolate should soon (summer 2014) become available for other research use through BEI Resources (Manassas, VA, USA).

**Figure 2:**
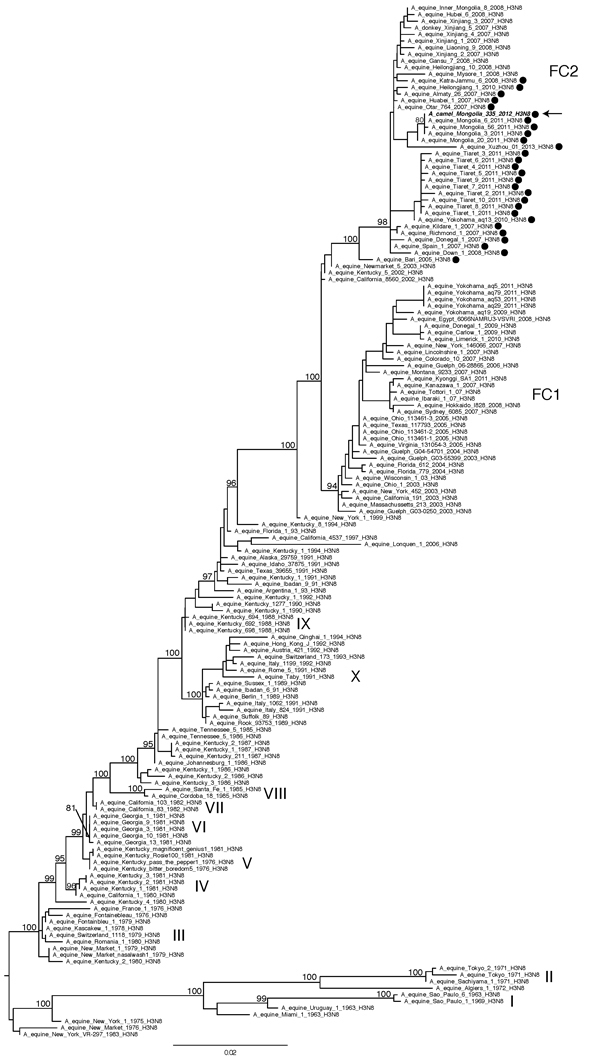
Evolutionary relationships of 155 full-length hemagglutinin sequences from equine A(H3N8)viruses collected globally and A/camel/Mongolia/335/2012 (arrow). The 2 clades associated with most recent equine influenza A(H3N8) viruses, Florida clade 1 and Florida clade 2, are denoted as FC1 and FC2, respectively, and with nomenclature adopted previously (*13*). The maximum-likelihood tree is midpoint rooted for clarity, and all branch lengths are drawn to scale. High (>70) bootstrap values are provided for key nodes. Hemagglutinin sequences containing a 2aa insertion are identified with a solid black circle. Scale bar indicates nucleotide substitutions per site.

For each of the 8 viral genome segments, phylogenetic trees were inferred separately by use of the maximum-likelihood methods available in RAxML version 7.2.6 (*12*), a general time-reversible model of nucleotide substitution, and a gamma-distributed rate variation among sites, with a bootstrapping resampling process (500 replicates). All 8 viral genome segments (polymerase basic protein [PB] 2, PB1, polymerase acidic protein (PA), hemagglutinin (HA) , nucleocapsid protein (NP), neuraminidase (NA), matrix protein (MP), and nonstructural protein (NS) are closely related to the equine influenza A(H3N8) viruses that have recently been circulating among the horse population in Asia; this lineage is evolutionarily distinct from the influenza A(H3N8) viruses circulating among birds in Asia (Technical Appendix Figures 1–7). A more detailed phylogenetic analysis of the hemagglutinin segment performed by using the additional background sequence data representing the global diversity of influenza A(H3N8) viruses in horses indicates that A/camel/Mongolia/335/2012 is positioned within Florida clade 2 (FC2) (Figure 2) (*13*) and, more specifically, within a bootstrap-supported clade that contains 3 influenza A(H3N8) viruses isolated from horses in Mongolia in 2011: A/equine/Mongolia/6/2011 (100% nt similarity), A/equine/Mongolia/56/2011 (99.9% nt similarity), and A/equine/Mongolia/3/2011 (100% nt similarity). A/camel/Mongolia/335/2012 also contains an insertion of 2 aa (I and F) near the beginning of the hemagglutinin sequence (hemagglutinin positions 8–9, Technical Appendix) that was first detected among FC2 equine viruses in 2005 (A/equine/Bari/2005/H3N8) and has been detected in most FC2 viruses, including all viruses that are closely related to A/camel/Mongolia/335/2012.

## Conclusions 

The phylogeny indicates that A/camel/Mongolia/335/2012 probably represents a relatively recent horse-to-camel transmission event. Without additional isolates from camels or corresponding epidemiologic data, and given the close genetic relationship between A/camel/Mongolia/335/2012 and related equine viruses, it is impossible to determine at this time whether the virus has been successfully transmitted from camel to camel.

In recent years, enhanced surveillance has detected influenza A viruses across a wider range of mammalian hosts, including horses, swine, dogs (*14*), seals (*15*), cats, and now camels, providing a more complete picture of the ecology of influenza A viruses beyond their presence in birds. How influenza A viruses successfully jump from 1 host species to another and what the constraints on interspecies transmission are remain key questions about influenza virus ecology and assessments of pandemic threats. Our findings highlight the need to further elucidate the ecology of influenza viruses and other pathogens in free-ranging camel populations.

Technical Appendix Tables showing viruses used in phylogenic analysis, and figures showing phylogenetic trees constructed by using full-length reads of 7 influenza A virus gene segments.
