# Supplementary material for: Equine Influenza A(H3N8) Virus Isolated from Bactrian Camel, Mongolia
Source: Emerg Infect Dis. 2014 Dec;20(12):2144–7. doi: 10.3201/eid2012.140435 (PMC4257804; doi:10.3201/eid2012.140435)
Supplement: Technical Appendix — Tables showing viruses used in phylogenic analysis, and figures showing phylogenetic trees constructed by using full-length reads of 7 influenza A virus gene segments. [file 14-0435-Techapp-s1.pdf]

Article DOI: http://dx.doi.org/10.3201/eid2012.140435

Equine Influenza A(H3N8) Virus Isolated from Bactrian Camel, Mongolia

Technical Appendix

Technical Appendix Table 1. Virus name, subtype, host species, collection date, and GenBank accession number (PB2 segment) for the 36 H3N8 viruses used in the whole-genome phylogenetic analysis conducted for all eight viral genome segments.

Virus name	Subtype	Host species	Collection date	Accession no.
A_camel_Mongolia_335_2012	H3N8	Camel	Nov-2012	CY164127.1
A_avian_Japan_8KI0102_2008	H3N8	Avian	Oct-08-08	CY079266
A_avian_Japan_8KI0129_2008	H3N8	Avian	Oct-08-08	CY079258
A_avian_Japan_8KI0150_2008	H3N8	Avian	Oct-08-08	CY079242
A_avian_Japan_8KI0162_2008	H3N8	Avian	Oct-08-08	CY079234
A_chicken_Laos_A0573_2007	H3N8	Avian	2007	CY040963
A_chicken_Vietnam_G14_2008	H3N8	Avian	Jan-2008	AB593452
A_donkey_Xinjiang_5_2007	H3N8	Equine	Dec-2007	EU794572
A_duck_Beijing_40_04	H3N8	Avian	2004	EU492488
A_duck_Beijing_61_05	H3N8	Avian	2005	EU492492
A_duck_Hokkaido_8_1980	H3N8	Avian	1980	AB274963
A_duck_Hunan_S1256_2012	H3N8	Avian	Mar-23-12	CY146601
A_duck_Hunan_S1824_2012	H3N8	Avian	Mar-24-12	CY146625
A_duck_Nanchang_1681_1992	H3N8	Avian	Dec-01-92	CY005475
A_duck_Vietnam_G119_2006	H3N8	Avian	Nov-2006	AB593428
A_environment_Hunan_S4350_2011	H3N8	Avian	Nov-13-11	CY146753
A_equine_Gansu_7_2008	H3N8	Equine	Jan-2008	EU794492
A_equine_Heilongjiang_1_2010_	H3N8	Equine	Apr-23-10	KF309031
A_equine_Heilongjiang_10_2008	H3N8	Equine	Apr-2008	EU794508
A_equine_Huabei_1_2007	H3N8	Equine	Dec-03-07	GU571147
A_equine_Inner_Mongolia_8_2008	H3N8	Equine	Feb-2008	EU794524
A_equine_Kyonggi_SA1_2011	H3N8	Equine	Jul-01-11	JX844143
A_equine_Liaoning_9_2008	H3N8	Equine	Apr-2008	EU794516
A_equine_Qinghai_1_1994	H3N8	Equine	1994	EU794532
A_equine_Sachiyama_1_1971	H3N8	Equine	1971	CY034941
A_equine_Tokyo_2_1971	H3N8	Equine	1971	CY096922
A_equine_Tottori_1_07	H3N8	Equine	2007	AB591847
A_equine_Xinjiang_1_2007	H3N8	Equine	Nov-2007	EU794540
A_equine_Xinjiang_2_2007	H3N8	Equine	Nov-2007	EU794548
A_equine_Xinjiang_3_2007	H3N8	Equine	Nov-2007	EU794556
A_equine_Xinjiang_4_2007	H3N8	Equine	Dec-2007	EU794564

Technical Appendix Table 1. Virus name, subtype, host species, collection date, and GenBank accession number (PB2 segment) for the 36 H3N8 viruses used in the whole-genome phylogenetic analysis conducted for all eight viral genome segments.

Virus name	Subtype	Host species	Collection date	Accession no.
A_equine_Xuzhou_01_2013	H3N8	Equine	Aug-27-13	KF806992
A_Mallard_SanJiang_90_2006_2006	H3N8	Avian	2006	CY100631
A_muscovy_duck_Vietnam_LBM240_2012	H3N8	Avian	2012	AB786912
A_swine_Anhui_01_2006	H3N8	Swine	Jan-06-06	FJ200417
A_swine_Chibi_01_2005	H3N8	Swine	Dec-15-05	FJ200425

Technical Appendix Table 2. Virus names for the hemagglutinin sequences of the 155 equine A/H3N8 viruses used in Figure 1. Viruses containing the two amino acid insertion near the beginning of the hemagglutinin are specified.

Virus name	Subtype	Insertion
A_camel_Mongolia_335_2012	H3N8	yes
A_donkey_Xinjiang_5_2007	H3N8	
A_equine_Alaska_29759_1991	H3N8	
A_equine_Algiers_1_1972	H3N8	
A_equine_Almaty_26_2007	H3N8	yes
A_equine_Argentina_1_93	H3N8	
A_equine_Austria_421_1992	H3N8	
A_equine_Bari_2005	H3N8	yes
A_equine_Berlin_1_1989	H3N8	
A_equine_California_1_1980	H3N8	
A_equine_California_103_1982	H3N8	
A_equine_California_191_2003	H3N8	
A_equine_California_4537_1997	H3N8	
A_equine_California_83_1982	H3N8	
A_equine_California_8560_2002	H3N8	
A_equine_Carlow_1_2009	H3N8	
A_equine_Colorado_10_2007	H3N8	
A_equine_Cordoba_18_1985	H3N8	
A_equine_Donegal_1_2007	H3N8	yes
A_equine_Donegal_1_2009	H3N8	
A_equine_Down_1_2008	H3N8	yes
A_equine_Egypt_6066NAMRU3-VSVRI_2008	H3N8	
A_equine_Florida_1_93	H3N8	
A_equine_Florida_612_2004	H3N8	
A_equine_Florida_779_2004	H3N8	
A_equine_Fontainbleu_1_1979	H3N8	
A_equine_Fontainebleau_1976	H3N8	
A_equine_France_1_1976	H3N8	
A_equine_Gansu_7_2008	H3N8	
A_equine_Georgia_1_1981	H3N8	
A_equine_Georgia_10_1981	H3N8	

Technical Appendix Table 2. Virus names for the hemagglutinin sequences of the 155 equine A/H3N8 viruses used in Figure 1. Viruses containing the two amino acid insertion near the beginning of the hemagglutinin are specified.

Virus name	Subtype	Insertion
A_camel_Mongolia_335_2012	H3N8	yes
A_equine_Georgia_13_1981	H3N8	
A_equine_Georgia_3_1981	H3N8	
A_equine_Georgia_9_1981	H3N8	
A_equine_Guelph_06-28865_2006	H3N8	
A_equine_Guelph_G03-0250_2003	H3N8	
A_equine_Guelph_G03-55399_2003	H3N8	
A_equine_Guelph_G04-54701_2004	H3N8	
A_equine_Heilongjiang_1_2010	H3N8	yes
A_equine_Heilongjiang_10_2008	H3N8	
A_equine_Hokkaido_I828_2008	H3N8	
A_equine_Hong_Kong_J_1992	H3N8	
A_equine_Huabei_1_2007	H3N8	yes
A_equine_Hubei_6_2008	H3N8	
A_equine_Ibadan_6_91	H3N8	
A_equine_Ibadan_9_91	H3N8	
A_equine_Ibaraki_1_07	H3N8	
A_equine_Idaho_37875_1991	H3N8	
A_equine_Inner_Mongolia_8_2008	H3N8	
A_equine_Italy_1062_1991	H3N8	
A_equine_Italy_1199_1992	H3N8	
A_equine_Italy_824_1991	H3N8	
A_equine_Johannesburg_1_1986	H3N8	
A_equine_Kanazawa_1_2007	H3N8	
A_equine_Kascakew_1_1978	H3N8	
A_equine_Katra-Jammu_6_2008	H3N8	yes
A_equine_Kentucky_1_1981	H3N8	
A_equine_Kentucky_1_1986	H3N8	
A_equine_Kentucky_1_1987	H3N8	
A_equine_Kentucky_1_1990	H3N8	
A_equine_Kentucky_1_1991	H3N8	
A_equine_Kentucky_1_1992	H3N8	
A_equine_Kentucky_1_1994	H3N8	
A_equine_Kentucky_1277_1990	H3N8	
A_equine_Kentucky_2_1980	H3N8	
A_equine_Kentucky_2_1981	H3N8	
A_equine_Kentucky_2_1986	H3N8	
A_equine_Kentucky_2_1987	H3N8	
A_equine_Kentucky_211_1987	H3N8	
A_equine_Kentucky_3_1981	H3N8	

Technical Appendix Table 2. Virus names for the hemagglutinin sequences of the 155 equine A/H3N8 viruses used in Figure 1. Viruses containing the two amino acid insertion near the beginning of the hemagglutinin are specified.

**Virus name**	**Subtype**	**Insertion**
A_camel_Mongolia_335_2012	H3N8	yes
A_equine_Kentucky_3_1986	H3N8	
A_equine_Kentucky_4_1980	H3N8	
A_equine_Kentucky_5_2002	H3N8	
A_equine_Kentucky_692_1988	H3N8	
A_equine_Kentucky_694_1988	H3N8	
A_equine_Kentucky_698_1988	H3N8	
A_equine_Kentucky_8_1994	H3N8	
A_equine_Kentucky_bitter_boredom5_1976	H3N8	
A_equine_Kentucky_magnificent_genius1_1981	H3N8	
A_equine_Kentucky_pass_the_pepper1_1976	H3N8	
A_equine_Kentucky_Rosie100_1981	H3N8	
A_equine_Kildare_1_2007	H3N8	yes
A_equine_Kyonggi_SA1_2011	H3N8	
A_equine_Liaoning_9_2008	H3N8	
A_equine_Limerick_1_2010	H3N8	
A_equine_Lincolnshire_1_2007	H3N8	
A_equine_Lonquen_1_2006	H3N8	
A_equine_Massachussetts_213_2003	H3N8	
A_equine_Miami_1_1963	H3N8	
A_equine_Mongolia_20_2011	H3N8	yes
A_equine_Mongolia_3_2011	H3N8	yes
A_equine_Mongolia_56_2011	H3N8	yes
A_equine_Mongolia_6_2011	H3N8	yes
A_equine_Montana_9233_2007	H3N8	
A_equine_Mysore_1_2008	H3N8	
A_equine_New_Market_1_1979	H3N8	
A_equine_New_Market_1976	H3N8	
A_equine_New_Market_nasalwash1_1979	H3N8	
A_equine_New_York_1_1975	H3N8	
A_equine_New_York_1_1999	H3N8	
A_equine_New_York_146066_2007	H3N8	
A_equine_New_York_452_2003	H3N8	
A_equine_New_York_VR-297_1983	H3N8	
A_equine_Newmarket_5_2003	H3N8	
A_equine_Ohio_1_2003	H3N8	
A_equine_Ohio_113461-1_2005	H3N8	
A_equine_Ohio_113461-2_2005	H3N8	
A_equine_Ohio_113461-3_2005	H3N8	
A_equine_Otar_764_2007	H3N8	yes

Technical Appendix Table 2. Virus names for the hemagglutinin sequences of the 155 equine A/H3N8 viruses used in Figure 1. Viruses containing the two amino acid insertion near the beginning of the hemagglutinin are specified.

**Virus name**	**Subtype**	**Insertion**
A_camel_Mongolia_335_2012	H3N8	yes
A_equine_Qinghai_1_1994	H3N8	
A_equine_Richmond_1_2007	H3N8	yes
A_equine_Romania_1_1980	H3N8	
A_equine_Rome_5_1991	H3N8	
A_equine_Rook_93753_1989	H3N8	
A_equine_Sachiyama_1_1971	H3N8	
A_equine_Santa_Fe_1_1985	H3N8	
A_equine_Sao_Paulo_1_1969	H3N8	
A_equine_Sao_Paulo_6_1963	H3N8	
A_equine_Spain_1_2007	H3N8	yes
A_equine_Suffolk_89	H3N8	
A_equine_Sussex_1_1989	H3N8	
A_equine_Switzerland_1118_1979	H3N8	
A_equine_Switzerland_173_1993	H3N8	
A_equine_Sydney_6085_2007	H3N8	
A_equine_Taby_1991	H3N8	
A_equine_Tennessee_5_1985	H3N8	
A_equine_Tennessee_5_1986	H3N8	
A_equine_Texas_117793_2005	H3N8	
A_equine_Texas_39655_1991	H3N8	
A_equine_Tiaret_1_2011	H3N8	yes
A_equine_Tiaret_10_2011	H3N8	yes
A_equine_Tiaret_2_2011	H3N8	yes
A_equine_Tiaret_3_2011	H3N8	yes
A_equine_Tiaret_4_2011	H3N8	yes
A_equine_Tiaret_5_2011	H3N8	yes
A_equine_Tiaret_6_2011	H3N8	yes
A_equine_Tiaret_7_2011	H3N8	yes
A_equine_Tiaret_8_2011	H3N8	yes
A_equine_Tiaret_9_2011	H3N8	yes
A_equine_Tokyo_1971	H3N8	
A_equine_Tokyo_2_1971	H3N8	
A_equine_Tottori_1_07	H3N8	
A_equine_Uruguay_1_1963	H3N8	
A_equine_Virginia_131054-3_2005	H3N8	
A_equine_Wisconsin_1_03	H3N8	
A_equine_Xinjiang_1_2007	H3N8	
A_equine_Xinjiang_2_2007	H3N8	
A_equine_Xinjiang_3_2007	H3N8	

Technical Appendix Table 2. Virus names for the hemagglutinin sequences of the 155 equine A/H3N8 viruses used in Figure 1. Viruses containing the two amino acid insertion near the beginning of the hemagglutinin are specified.

Virus name	Subtype	Insertion
A_camel_Mongolia_335_2012	H3N8	yes
A_equine_Xinjiang_4_2007	H3N8	
A_equine_Xuzhou_01_2013	H3N8	yes
A_equine_Yokohama_aq13_2010	H3N8	yes
A_equine_Yokohama_aq19_2009	H3N8	
A_equine_Yokohama_aq29_2011	H3N8	
A_equine_Yokohama_aq5_2011	H3N8	
A_equine_Yokohama_aq53_2011	H3N8	
A_equine_Yokohama_aq79_2011	H3N8	

Technical Appendix Table 3. Position of the two amino acid insertion at the beginning of the hemagglutinin sequence.

Example of virus with insertion:	
A/equine/Almaty/26/2007/H3N8	MKTTIIFIFILLTHW
Example of virus without insertion:	
A/equine/Alaska/29759/1991/H3N8	MKTTIIL--ILLTHW

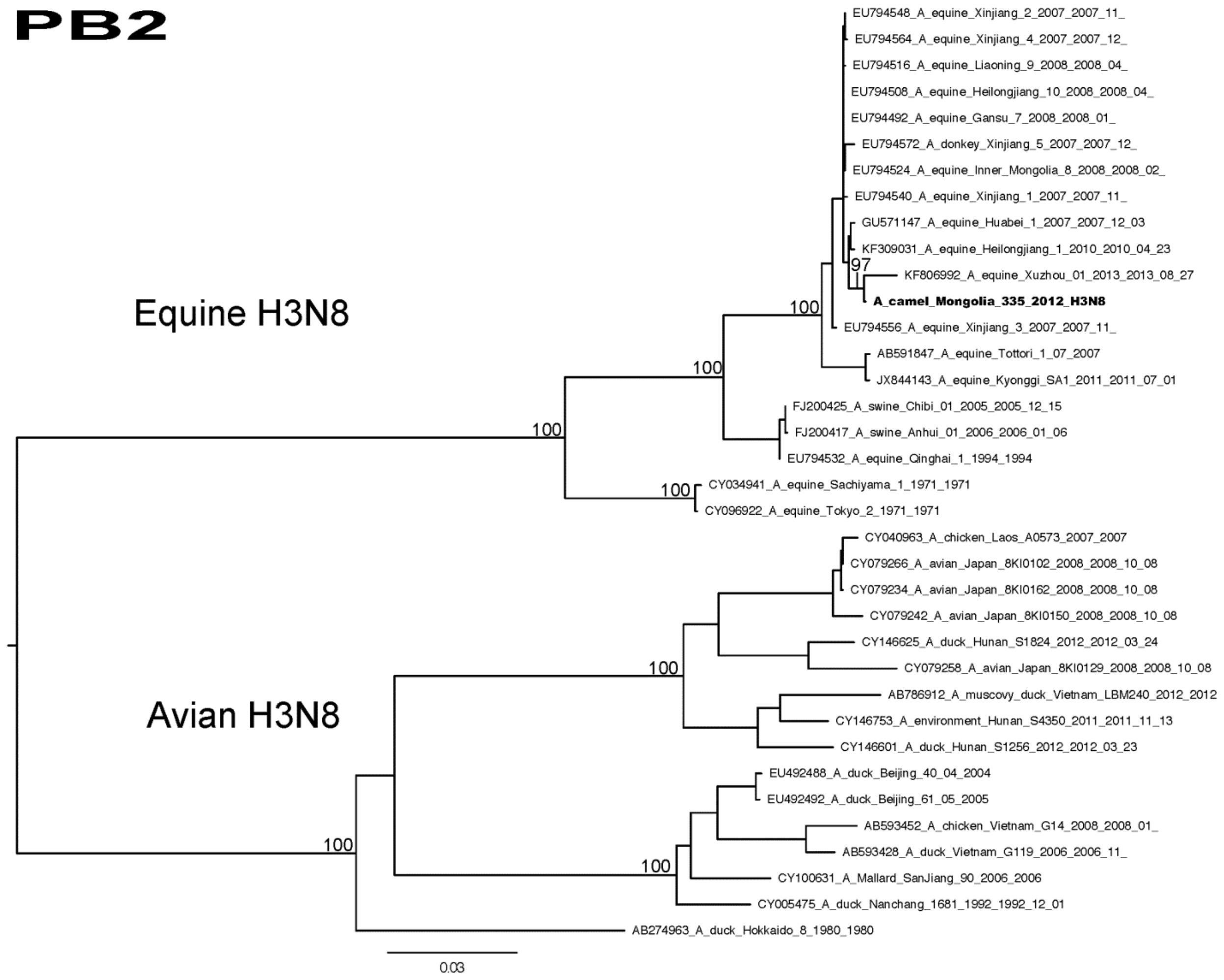

Technical Appendix Figure 1. Evolutionary relationships of the PB2 segment of 36 influenza A viruses of the H3N8 subtype collected in Asia from horses (n = 17), avian species (n = 16), swine (n = 2), and a camel (A/camel/Mongolia/335/2012(H3N8), highlighted in bold). The tree is midpoint rooted for clarity, and all branch lengths are drawn to scale. High bootstrap values (> 70) are provided for key nodes. Scale bar indicates nucleotide substitutions per site.

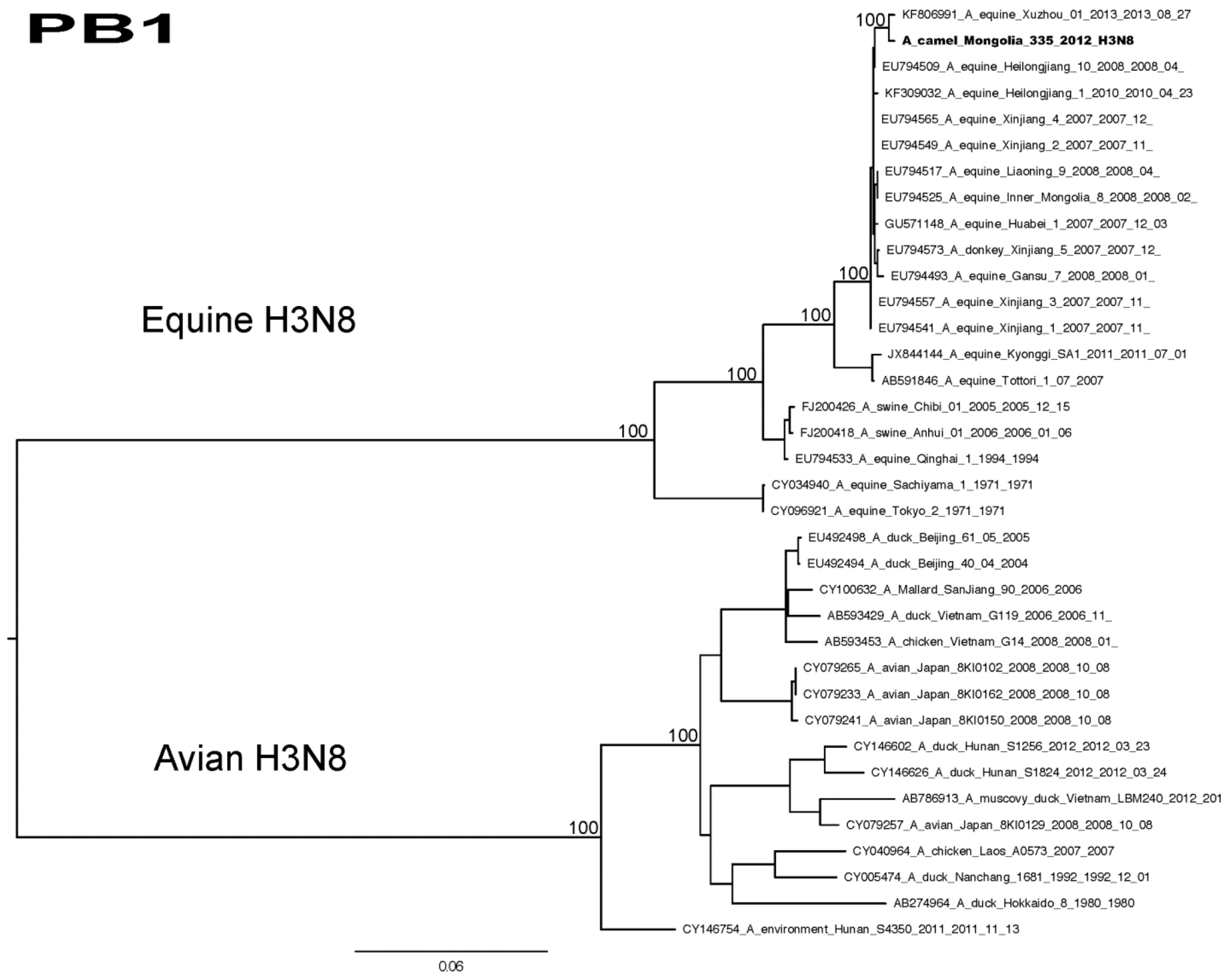

Technical Appendix Figure 2. Evolutionary relationships of the PB1 segment of 36 influenza A viruses of the H3N8 subtype collected in Asia from horses (n = 17), avian species (n = 16), swine (n = 2), and a camel (A/camel/Mongolia/335/2012(H3N8), highlighted in bold). The tree is midpoint rooted for clarity, and all branch lengths are drawn to scale. High bootstrap values (> 70) are provided for key nodes. Scale bar indicates nucleotide substitutions per site.

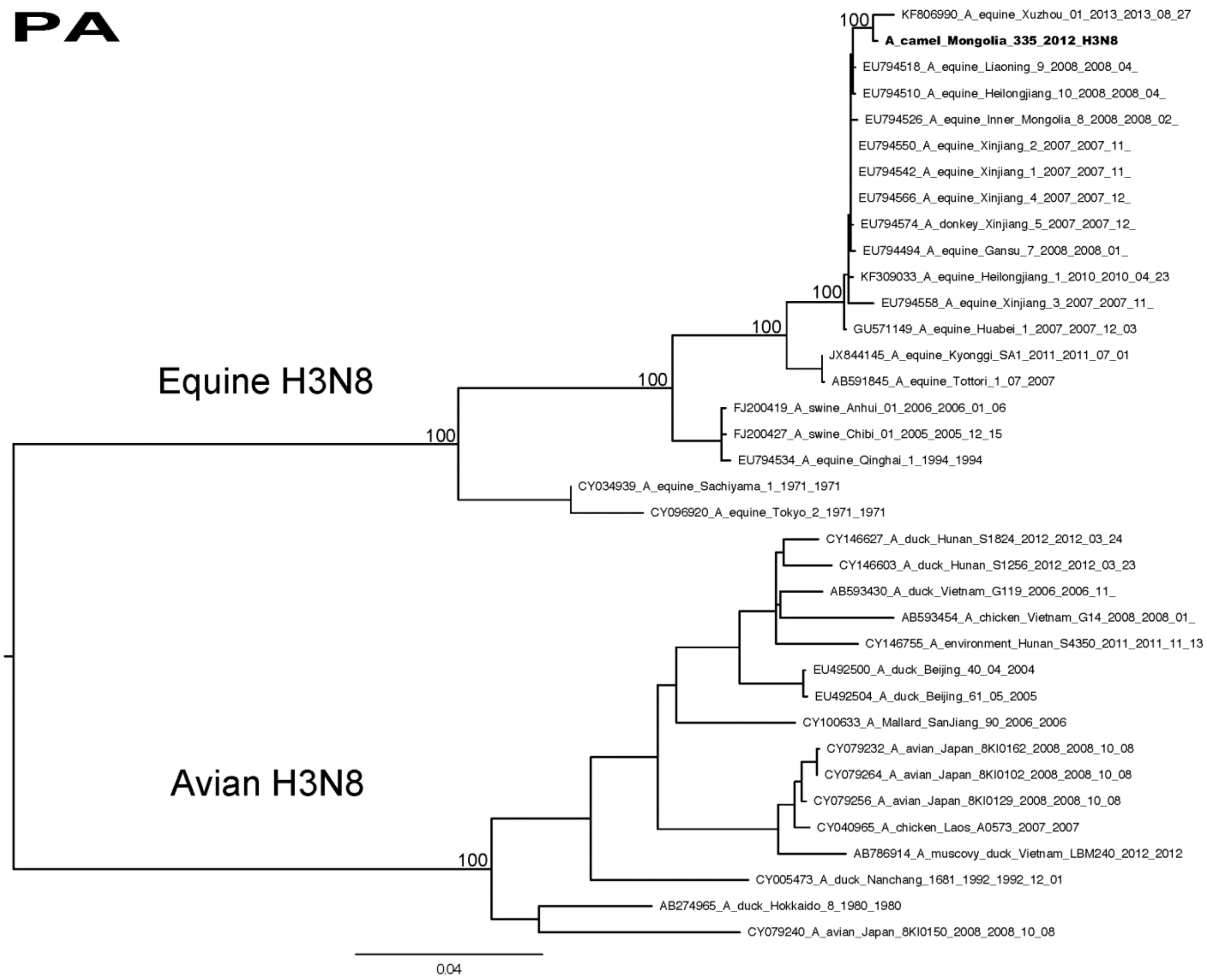

Technical Appendix Figure 3. Evolutionary relationships of the PA segment of 36 influenza A viruses of the H3N8 subtype collected in Asia from horses (n = 17), avian species (n = 16), swine (n = 2), and a camel (A/camel/Mongolia/335/2012(H3N8), highlighted in bold). The tree is midpoint rooted for clarity, and all branch lengths are drawn to scale. High bootstrap values (> 70) are provided for key nodes. Scale bar indicates nucleotide substitutions per site.

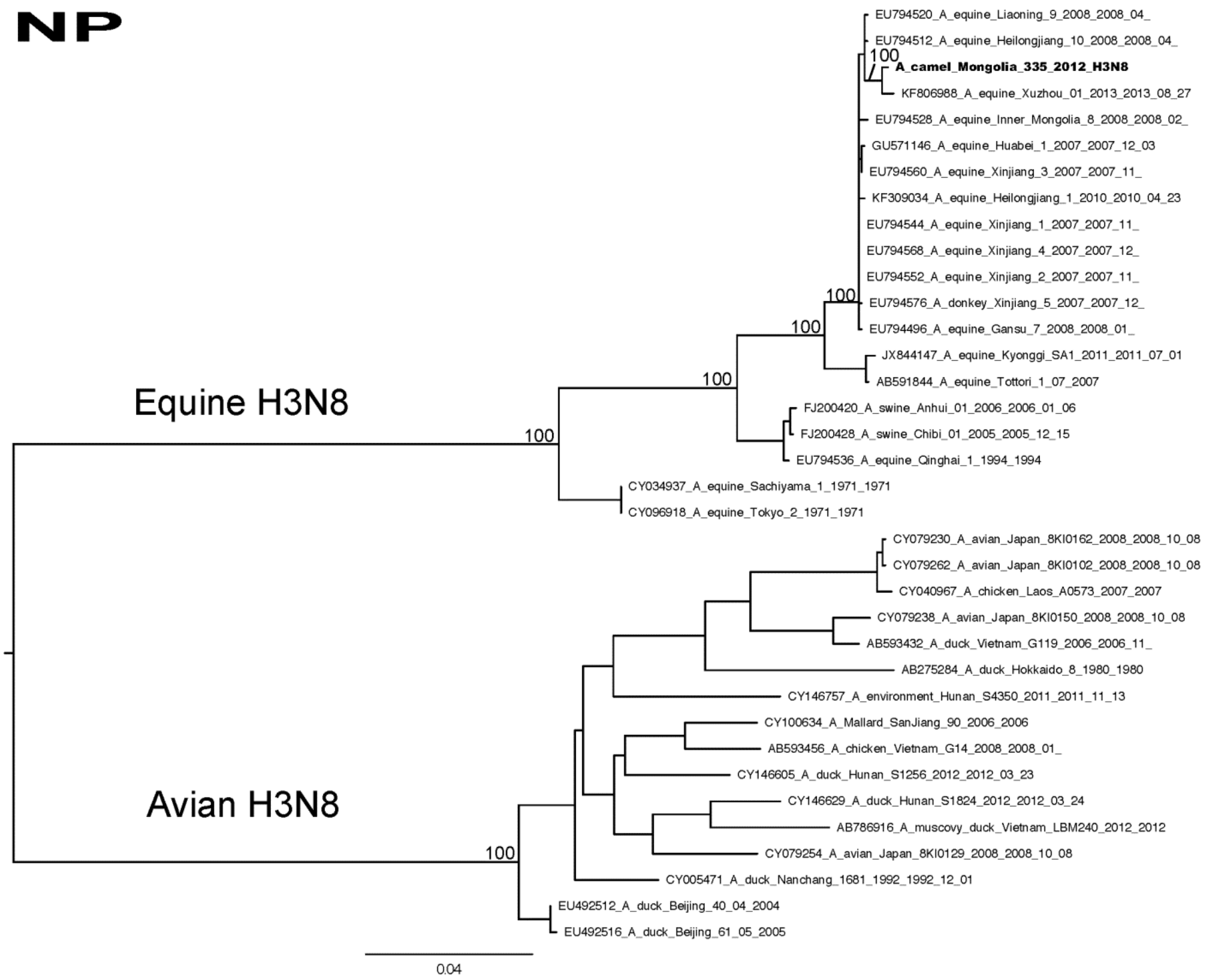

Technical Appendix Figure 4. Evolutionary relationships of the NP segment of 36 influenza A viruses of the H3N8 subtype collected in Asia from horses (n = 17), avian species (n = 16), swine (n = 2), and a camel (A/camel/Mongolia/335/2012(H3N8), highlighted in bold). The tree is midpoint rooted for clarity, and all branch lengths are drawn to scale. High bootstrap values (> 70) are provided for key nodes. Scale bar indicates nucleotide substitutions per site.

NA

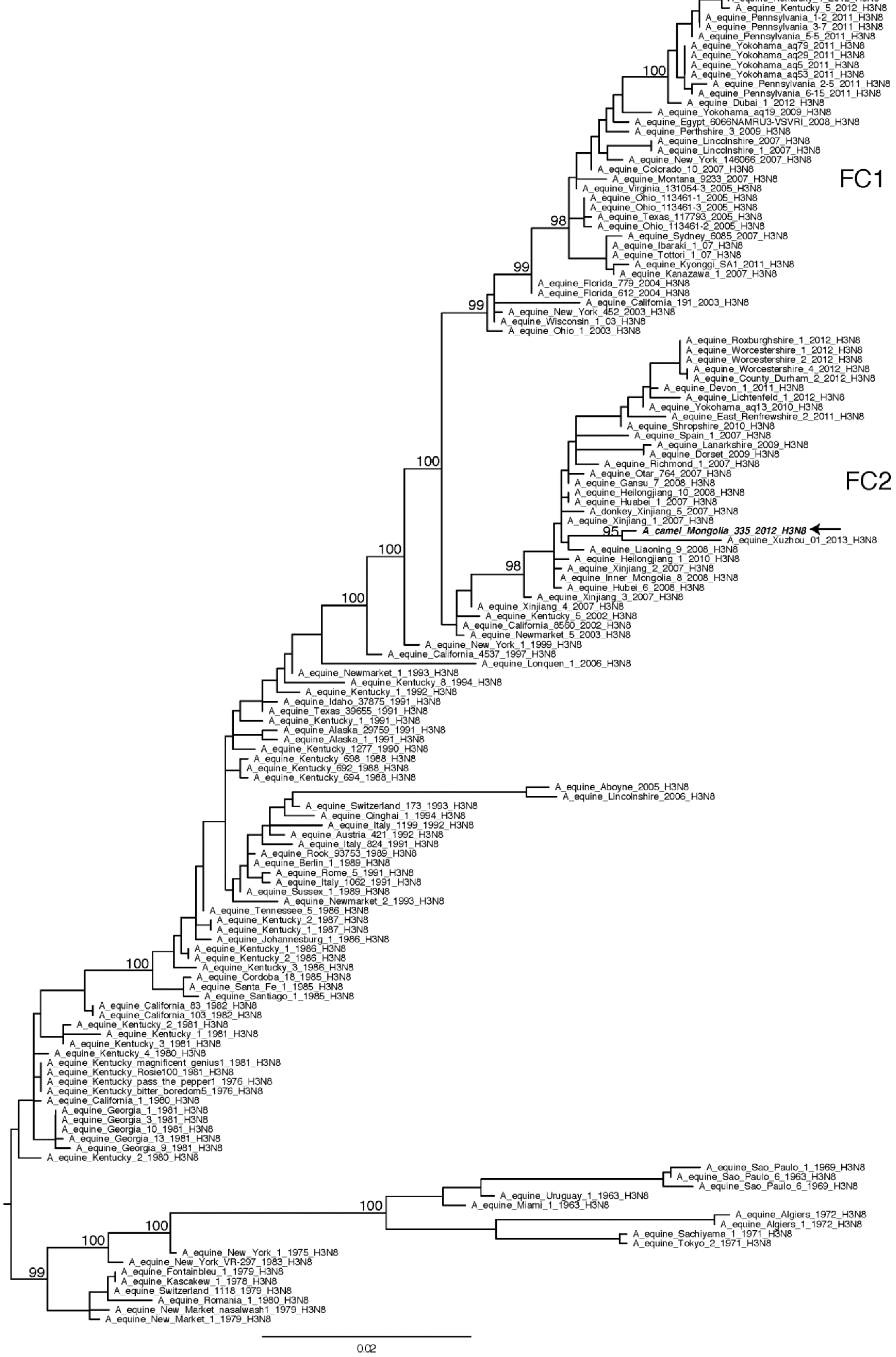

A_equine_Kentucky_2_2012_H3N8
A_equine_Kentucky_3_2012_H3N8
A_equine_Kentucky_4_2012_H3N8
A_equine_Kentucky_5_2012_H3N8
A_equine_Pennsylvania_1-2_2011_H3N8
A_equine_Pennsylvania_3-7_2011_H3N8
A_equine_Pennsylvania_5-5_2011_H3N8
A_equine_Yokohama_aq79_2011_H3N8
A_equine_Yokohama_aq29_2011_H3N8
A_equine_Yokohama_aq5_2011_H3N8
A_equine_Yokohama_aq53_2011_H3N8
A_equine_Pennsylvania_2-5_2011_H3N8
A_equine_Pennsylvania_6-15_2011_H3N8
A_equine_Dubai_1_2012_H3N8
A_equine_Yokohama_aq19_2009_H3N8
A_equine_Egypt_6066NAMRU3-VSVRI_2008_H3N8
A_equine_Perthshire_3_2009_H3N8
A_equine_Lincolnshire_2007_H3N8
A_equine_Lincolnshire_1_2007_H3N8
A_equine_New_York_146066_2007_H3N8
A_equine_Colorado_10_2007_H3N8
A_equine_Montana_9233_2007_H3N8
A_equine_Virginia_131054-3_2005_H3N8
A_equine_Ohio_113461-1_2005_H3N8
A_equine_Ohio_113461-3_2005_H3N8
A_equine_Texas_117793_2005_H3N8
A_equine_Ohio_113461-2_2005_H3N8
A_equine_Sydney_6085_2007_H3N8
A_equine_Ibaraki_1_07_H3N8
A_equine_Tottori_1_07_H3N8
A_equine_Kyonggi_SA1_2011_H3N8
A_equine_Kanazawa_1_2007_H3N8
A_equine_Florida_779_2004_H3N8
A_equine_Florida_612_2004_H3N8
A_equine_California_191_2003_H3N8
A_equine_New_York_452_2003_H3N8
A_equine_Wisconsin_1_03_H3N8
A_equine_Ohio_1_2003_H3N8
FC1
100
98
99
99
A_equine_Roxburghshire_1_2012_H3N8
A_equine_Worcestershire_1_2012_H3N8
A_equine_Worcestershire_2_2012_H3N8
A_equine_Worcestershire_4_2012_H3N8
A_equine_County_Durham_2_2012_H3N8
A_equine_Devon_1_2011_H3N8
A_equine_Lichtenfeld_1_2012_H3N8
A_equine_Yokohama_aq13_2010_H3N8
A_equine_East_Renfrewshire_2_2011_H3N8
A_equine_Shropshire_2010_H3N8
A_equine_Spain_1_2007_H3N8
A_equine_Lanarkshire_2009_H3N8
A_equine_Dorset_2009_H3N8
A_equine_Richmond_1_2007_H3N8
A_equine_Otar_764_2007_H3N8
A_equine_Gansu_7_2008_H3N8
A_equine_Heilongjiang_10_2008_H3N8
A_equine_Huabei_1_2007_H3N8
A_donkey_Xinjiang_5_2007_H3N8
A_equine_Xinjiang_1_2007_H3N8
95
A_camel_Mongolia_335_2012_H3N8
A_equine_Xuzhou_01_2013_H3N8
A_equine_Liaoning_9_2008_H3N8
A_equine_Heilongjiang_1_2010_H3N8
A_equine_Xinjiang_2_2007_H3N8
A_equine_Inner_Mongolia_8_2008_H3N8
A_equine_Hubei_6_2008_H3N8
A_equine_Xinjiang_3_2007_H3N8
A_equine_Xinjiang_4_2007_H3N8
A_equine_Kentucky_5_2002_H3N8
A_equine_California_8560_2002_H3N8
A_equine_Newmarket_5_2003_H3N8
A_equine_New_York_1_1999_H3N8
A_equine_California_4537_1997_H3N8
A_equine_Lonquen_1_2006_H3N8
FC2
98
100
100
100
100
A_equine_Newmarket_1_1993_H3N8
A_equine_Kentucky_8_1994_H3N8
A_equine_Kentucky_1_1992_H3N8
A_equine_Idaho_37875_1991_H3N8
A_equine_Texas_39655_1991_H3N8
A_equine_Kentucky_1_1991_H3N8
A_equine_Alaska_29759_1991_H3N8
A_equine_Alaska_1_1991_H3N8
A_equine_Kentucky_1277_1990_H3N8
A_equine_Kentucky_698_1988_H3N8
A_equine_Kentucky_692_1988_H3N8
A_equine_Kentucky_694_1988_H3N8
A_equine_Aboyne_2005_H3N8
A_equine_Lincolnshire_2006_H3N8
A_equine_Switzerland_173_1993_H3N8
A_equine_Qinghai_1_1994_H3N8
A_equine_Italy_1199_1992_H3N8
A_equine_Austria_421_1992_H3N8
A_equine_Italy_824_1991_H3N8
A_equine_Rook_93753_1989_H3N8
A_equine_Berlin_1_1989_H3N8
A_equine_Rome_5_1991_H3N8
A_equine_Italy_1062_1991_H3N8
A_equine_Sussex_1_1989_H3N8
A_equine_Newmarket_2_1993_H3N8
A_equine_Tennessee_5_1986_H3N8
A_equine_Kentucky_2_1987_H3N8
A_equine_Kentucky_1_1987_H3N8
A_equine_Johannesburg_1_1986_H3N8
A_equine_Kentucky_1_1986_H3N8
A_equine_Kentucky_2_1986_H3N8
A_equine_Kentucky_3_1986_H3N8
A_equine_Cordoba_18_1985_H3N8
A_equine_Santa_Fe_1_1985_H3N8
A_equine_Santiago_1_1985_H3N8
100
A_equine_California_83_1982_H3N8
A_equine_California_103_1982_H3N8
A_equine_Kentucky_2_1981_H3N8
A_equine_Kentucky_1_1981_H3N8
A_equine_Kentucky_3_1981_H3N8
A_equine_Kentucky_4_1980_H3N8
A_equine_Kentucky_magnificent_genius1_1981_H3N8
A_equine_Kentucky_Rosie100_1981_H3N8
A_equine_Kentucky_pass_the_pepper1_1976_H3N8
A_equine_Kentucky_bitter_boredom5_1976_H3N8
A_equine_California_1_1980_H3N8
A_equine_Georgia_1_1981_H3N8
A_equine_Georgia_3_1981_H3N8
A_equine_Georgia_10_1981_H3N8
A_equine_Georgia_13_1981_H3N8
A_equine_Georgia_9_1981_H3N8
A_equine_Kentucky_2_1980_H3N8
A_equine_Sao_Paulo_1_1969_H3N8
A_equine_Sao_Paulo_6_1963_H3N8
A_equine_Sao_Paulo_6_1969_H3N8
A_equine_Uruguay_1_1963_H3N8
A_equine_Miami_1_1963_H3N8
A_equine_Algiers_1972_H3N8
A_equine_Algiers_1_1972_H3N8
A_equine_Sachiyama_1_1971_H3N8
A_equine_Tokyo_2_1971_H3N8
100
100
100
99
A_equine_New_York_1_1975_H3N8
A_equine_New_York_VR-297_1983_H3N8
A_equine_Fontainbleu_1_1979_H3N8
A_equine_Kascakew_1_1978_H3N8
A_equine_Switzerland_1118_1979_H3N8
A_equine_Romania_1_1980_H3N8
A_equine_New_Market_nasalwash1_1979_H3N8
A_equine_New_Market_1_1979_H3N8
0.02

Technical Appendix Figure 5. Evolutionary relationships of the NA segment of 142 influenza A viruses of the H3N8 subtype collected from horses and A/camel/Mongolia/335/2012(H3N8), highlighted in bold. The tree is midpoint rooted for clarity, and all branch lengths are drawn to scale. High bootstrap values (> 70) are provided for key nodes. Scale bar indicates nucleotide substitutions per site.

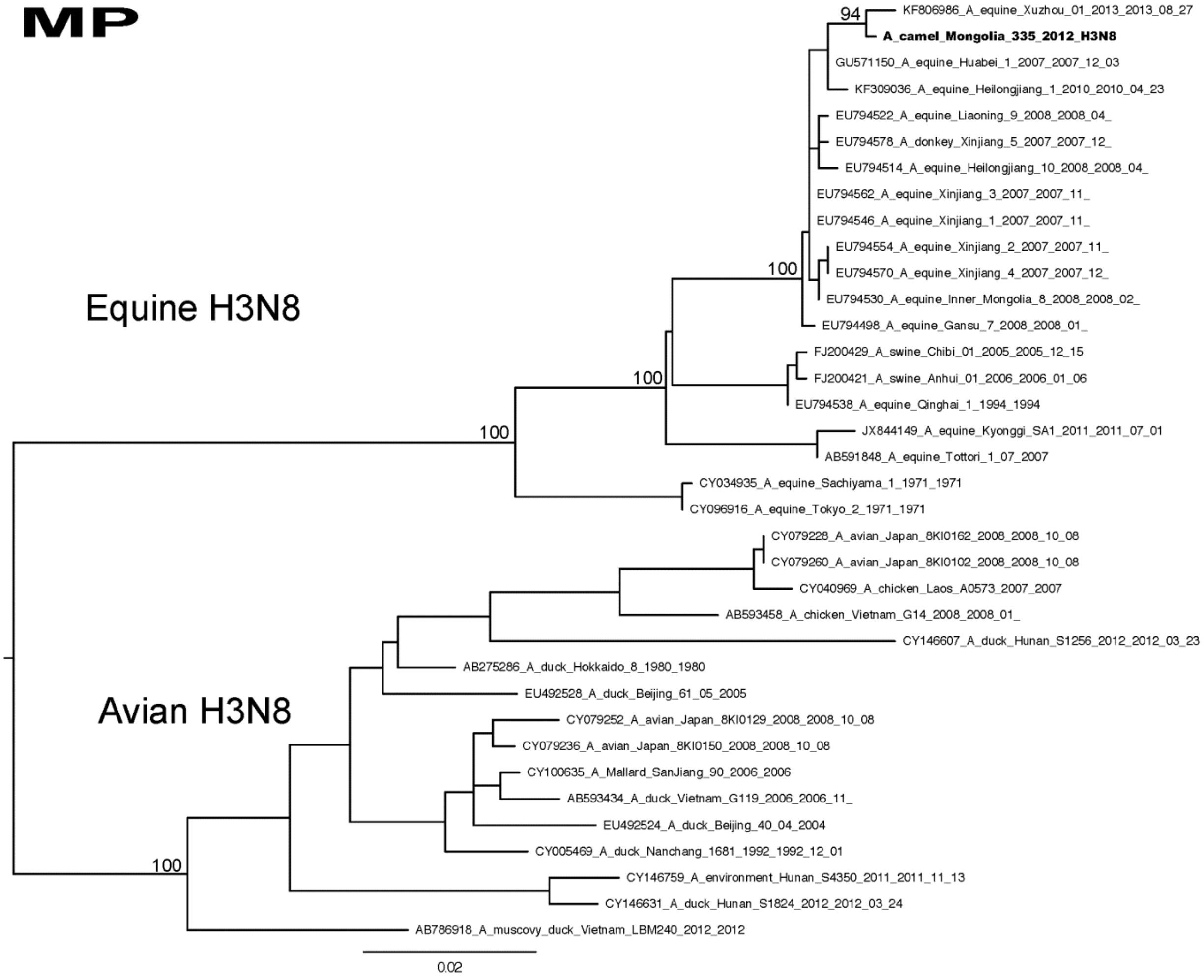

Technical Appendix Figure 6. Evolutionary relationships of the MP segment of 36 influenza A viruses of the H3N8 subtype collected in Asia from horses (n = 17), avian species (n = 16), swine (n = 2), and a camel (A/camel/Mongolia/335/2012(H3N8), highlighted in bold). The tree is midpoint rooted for clarity, and all branch lengths are drawn to scale. High bootstrap values (> 70) are provided for key nodes. Scale bar indicates nucleotide substitutions per site.

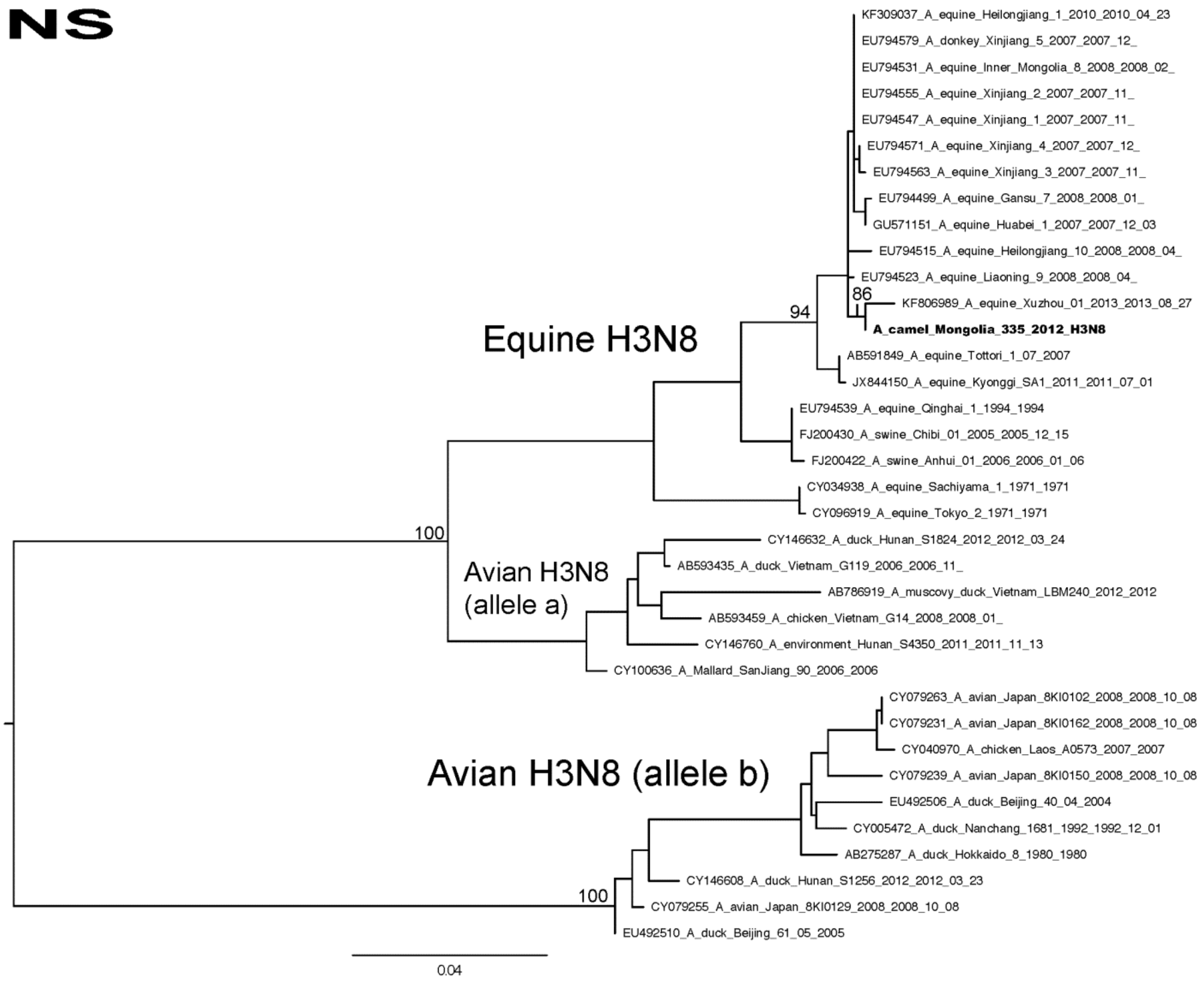

Technical Appendix Figure 7. Evolutionary relationships of the NS segment of 36 influenza A viruses of the H3N8 subtype collected in Asia from horses (n = 17), avian species (n = 16), swine (n = 2), and a camel (A/camel/Mongolia/335/2012(H3N8), highlighted in bold). The tree is midpoint rooted for clarity, and all branch lengths are drawn to scale. High bootstrap values (> 70) are provided for key nodes.
